# RAD51 in Breast Cancer: From Vulnerability to Resistance

**DOI:** 10.14740/wjon2764

**Published:** 2026-06-25

**Authors:** Rana Salman Anjum, Kazuaki Takabe

**Affiliations:** a Department of Oral Oncology, Roswell Park Comprehensive Cancer Center, Buffalo, NY 14263, USA; b Department of Surgical Oncology, Roswell Park Comprehensive Cancer Center, Buffalo, NY 14263, USA; c Department of Immunology, Roswell Park Comprehensive Cancer Center, Buffalo, NY 14263, USA; d Department of Surgery, University at Buffalo Jacobs School of Medicine and Biomedical Sciences the State University of New York, Buffalo, NY 14263, USA; e Department of Breast Surgery and Oncology, Tokyo Medical University, Tokyo 160-8402, Japan; f Department of Gastroenterological Surgery, Yokohama City University Graduate School of Medicine, Kanagawa 236-0004, Japan; g Division of Digestive and General Surgery, Niigata University Graduate School of Medical and Dental Sciences, Niigata 951-8520, Japan; h Department of Breast Surgery, Fukushima Medical University School of Medicine, Fukushima 960-1295, Japan

**Keywords:** RAD51, BRCA2, Homologous recombination, Replication fork, Breast cancer

## Abstract

Homologous recombination deficiency (HRD) has transformed the therapeutic landscape of breast cancer through the clinical success of poly(ADP-ribose) polymerase (PARP) inhibitors and platinum-based chemotherapy. Central to this vulnerability is radiation sensitivity 51 (RAD51), the recombinase that executes homologous DNA repair and stabilizes stalled replication forks. In breast cancer susceptibility type 1 (BRCA1)- and breast cancer susceptibility type 2 (BRCA2)-mutant tumors, impaired RAD51 loading produces profound sensitivity to DNA-damaging agents. However, accumulating evidence indicates that restoration of RAD51 function—particularly its role in replication fork protection—is an important mechanism underlying therapeutic resistance. Beyond its canonical role in strand exchange–mediated double-strand break (DSB) repair, RAD51 orchestrates replication fork reversal, stabilization, and restart under conditions of oncogene-driven replication stress. These fork-associated functions can be mechanistically separable from classical homologous recombination and may be sufficient to confer resistance to PARP inhibitors even in tumors with persistent genomic scar signatures. Thus, breast cancer evolution under therapeutic pressure can be conceptualized as a transition from RAD51 deficiency–driven vulnerability to RAD51-dependent adaptive survival. In this review, we integrate structural, mechanistic, and translational insights into RAD51 biology and propose a dynamic framework in which replication fork protection represents a central adaptive axis in resistant breast cancer. We discuss functional biomarkers of RAD51 activity, subtype-specific dependency patterns, and emerging strategies to therapeutically target RAD51 in PARP inhibitor–refractory and replication stress–high disease. Understanding when RAD51 is deficient and when it becomes indispensable will be critical for refining precision oncology approaches in breast cancer. We argue that future precision oncology strategies must move beyond static HRD classification toward dynamic assessment of RAD51-dependent replication stress tolerance.

## RAD51 as an Evolutionarily Conserved Genome Stabilizer and a Therapeutic Fulcrum in Breast Cancer

Homologous recombination (HR) is among the most evolutionarily conserved genome maintenance pathways in biology. From bacterial recombinase A (RecA) to archaeal RadA and mammalian RAD51, adenosine triphosphate (ATP)-dependent recombinases assemble on single-stranded DNA (ssDNA) to catalyze homology search and strand exchange [1–3]. For decades, this strand exchange reaction defined the biological identity of HR. However, recent mechanistic and translational advances compel a reassessment of RAD51’s role, particularly in cancer [4, 5].

In mammalian cells, RAD51 is no longer understood solely as the executor of high-fidelity double-strand break (DSB) repair. Instead, RAD51 functions at the intersection of DNA damage signaling, replication fork remodeling, chromatin architecture, and therapeutic adaptation [4, 6–8]. In breast cancer, this duality has profound implications. Loss of RAD51 loading underlies the vulnerability of BRCA-mutant tumors to poly(ADP-ribose) polymerase (PARP) inhibitors [9–11]. Yet restoration of RAD51-dependent replication fork protection emerges as an important mechanism of resistance [5, 12, 13]. Thus, RAD51 is both a tumor suppressor and an adaptive survival factor.

We propose that the clinical trajectory of breast cancer under DNA-damaging therapy is governed not simply by homologous recombination deficiency (HRD), but by the dynamic state of RAD51-dependent replication fork protection [4, 5, 12]. This conceptual shift reframes therapeutic targeting strategies and biomarker development.

### The recombinase core

RecA-family recombinases share a conserved ATPase core that drives nucleoprotein filament assembly [1–3, 14]. ATP binding promotes formation of an extended filament conformation competent for homology search; ATP hydrolysis regulates filament turnover [14–16]. This architecture has remained fundamentally conserved across three domains of life.

Yet regulatory complexity has expanded dramatically in eukaryotes. Human RAD51 operates within chromatinized genomes and is tightly coordinated by BRCA1, PALB2, BRCA2, RAD51 paralogs, checkpoint kinases, and ubiquitin ligases [17, 18]. This regulatory layering enables spatial and temporal control but also introduces vulnerabilities exploited during tumorigenesis.

### Beyond strand exchange: the emerging centrality of replication fork protection

The canonical pathway proceeds through DSB recognition by the Mre11, Rad50 and Nbs1 (MRN) complex followed by ataxia telangiectasia mutated (ATM) activation and phosphorylated histone H2AX (γH2AX) spreading, DNA end resection to generate 3′ ssDNA tails coated by replication protein A (RPA), and BRCA2-mediated RAD51 loading [17, 19]. RAD51 filament-driven homology search and strand invasion initiate DNA synthesis and intermediate resolution to restore chromosome integrity [19].

Inherited mutations disrupting BRCA1 or BRCA2 impair RAD51 loading and produce HRD, predisposing to breast cancer [9–11]. The therapeutic success of PARP inhibitors validated this model: HR-deficient tumors are selectively sensitive to replication-associated DNA lesions [9–11].

**Figure 1:**
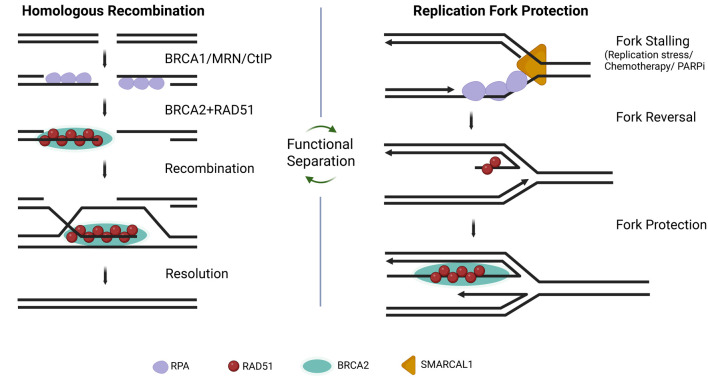
RAD51-mediated homologous recombination versus replication fork protection. The left panel illustrates classical homologous recombination repair initiated following DNA double-strand break formation and BRCA1/MRN/CtIP-mediated end resection, followed by BRCA2-dependent RAD51 loading and recombination-mediated repair. The right panel illustrates replication fork stalling, fork reversal, and RAD51-mediated fork protection during replication stress. Functional separation between homologous recombination repair and replication fork stabilization may contribute to therapeutic resistance in breast cancer.

However, canonical HR does not fully explain acquired resistance. Increasing evidence indicates that RAD51’s role at stalled replication forks—specifically fork reversal and protection of nascent DNA from nucleolytic degradation—is mechanistically separable from classical strand exchange [4, 6–8]. This distinction is clinically crucial. Tumors that restore fork protection may regain survival capacity even without fully restoring homologous DSB repair [5, 12, 13]. Figure 1 summarizes the conceptual distinction between RAD51-mediated HR repair and RAD51-dependent replication fork protection, highlighting the emerging separation-of-function framework underlying therapeutic resistance.

### RAD51 as a dynamic node in tumor evolution

The clinical trajectory of breast cancer can be understood through dynamic shifts in RAD51 activity rather than static classifications of HR proficiency. In the earliest phase, HRD arises from impaired RAD51 loading, most commonly due to BRCA1 or BRCA2 mutation [9–11]. This deficiency promotes genomic instability while simultaneously creating therapeutic vulnerability. Under sustained therapeutic pressure, tumor cells frequently acquire mechanisms that partially restore RAD51 function. These include BRCA reversion mutations, loss of 53BP1-mediated resection blockade, and re-establishment of replication fork stabilization [5, 12, 13, 20, 21]. Clinically, this transition manifests as acquired resistance, often accompanied by reappearance of RAD51 nuclear foci [5, 12].

With continued replication stress and treatment exposure, tumors may progress to a state of RAD51 dependency in which survival becomes contingent upon RAD51-mediated replication fork protection [4, 6–8]. Mechanistic studies demonstrate that RAD51 stabilizes reversed replication forks and protects nascent DNA from nuclease-mediated degradation independently of complete DSB repair [4, 6, 7]. In this context, the recombinase that was once absent and therapeutically exploitable becomes an adaptive survival factor.

## Structural and Mechanistic Architecture of RAD51: Filament Dynamics as a Regulatory Axis

RAD51 belongs to the RecA superfamily of P-loop ATPases, whose defining property is ATP-dependent polymerization on ssDNA [1–3]. Across bacteria, archaea, and eukaryotes, recombinase activity is executed by a nucleoprotein filament rather than an isolated monomeric enzyme [1–3, 14, 15]. In mammals, however, this filament has evolved into a tightly regulated, chromatin-integrated molecular assembly whose biological output depends less on catalytic chemistry and more on assembly dynamics [14, 15]. This distinction—“chemistry versus architecture”—is central to understanding RAD51 in breast cancer.

### The conserved catalytic engine: ATP-driven polymerization

RAD51 monomers contain a conserved ATP-binding core characteristic of RecA-family recombinases [14, 16]. ATP binding promotes assembly of an extended nucleoprotein filament competent for DNA pairing and recombination, whereas ATP hydrolysis regulates filament turnover and remodeling [1, 14, 15].

Structurally, nucleotide binding stabilizes inter-subunit contacts and positions DNA-binding loops for interaction with ssDNA [22, 23]. Filament assembly is therefore intrinsically cooperative and allosterically controlled. In mammalian cells, regulated filament assembly rather than catalytic chemistry alone appears central to RAD51 function in replication stress responses and therapeutic adaptation [24].

### Nucleation: the critical regulatory bottleneck

*In vivo*, the rate-limiting step of RAD51 function is not strand exchange chemistry but nucleation on RPA-coated ssDNA. RPA binds ssDNA with high affinity immediately after DNA end resection or fork stalling [17]. For RAD51 to assemble, RPA must be displaced—an energetically unfavorable exchange without mediator assistance.

BRCA2, through its BRC repeats, delivers RAD51 monomers directly to ssDNA and stabilizes early nucleation clusters [22, 25–27]. Single-molecule studies demonstrate that productive nucleation requires small clusters of RAD51 monomers (typically 2–5), after which cooperative filament extension proceeds rapidly [28, 29].

This kinetic bottleneck provides a powerful regulatory checkpoint. Modest perturbations in mediator function dramatically alter HR efficiency, making nucleation an attractive therapeutic intervention point.

### Filament topology and DNA stretching

The active RAD51 filament forms an extended helical nucleoprotein structure that promotes homology search and DNA pairing [14, 23]. ATP-bound filaments are structurally stable and recombination competent, whereas ADP-bound filaments are more prone to disassembly [15, 23]. This dynamic regulation of filament stability is important not only for HR but also for replication fork protection, where RAD51 filament stabilization contributes to protection of reversed forks from nucleolytic degradation [4, 6].

### Homology search: a probabilistic sampling process

RAD51 filaments promote homology search through transient interactions with duplex DNA, enabling identification of homologous sequences required for strand invasion and repair [29–31]. Efficient and tightly regulated homology recognition is potentially important for maintaining genome integrity. In therapy-resistant tumors, restoration of RAD51 filament assembly may therefore support both recombination-mediated repair and adaptive responses to replication stress [5, 12, 13].

### RAD54 and motor-coupled remodeling

RAD54, a SWI2/SNF2 family DNA translocase, acts downstream of RAD51 filament assembly. It stabilizes D-loops, promotes branch migration, and facilitates removal of RAD51 from double-stranded DNA following strand invasion [32–34]. RAD54-mediated remodeling couples recombination to DNA synthesis by clearing RAD51 from newly formed heteroduplex DNA. Thus, RAD51 must assemble efficiently but also be cleared efficiently. Dysregulated disassembly can generate toxic intermediates or replication interference [33].

### Filament disassembly: controlled termination of activity

RAD51 disassembly is governed by intrinsic ATP hydrolysis [15], RAD54-mediated translocation [33], and anti-recombinase helicases such as F-box DNA helicase 1 (FBH1) [35]. FBH1 promotes filament destabilization and limits excessive recombination. In cancer cells experiencing elevated replication stress, stabilization of RAD51 filaments at stalled forks may be advantageous [4, 6]. However, excessive stabilization can promote genomic rearrangements. Filament dynamics therefore represent a finely tuned balance between protective and pathological outcomes.

### Mediator interfaces as druggable surfaces

Structural analyses of the BRCA2–RAD51 interaction revealed that BRC repeats bind a hydrophobic pocket overlapping RAD51 oligomerization interfaces [22, 26]. This interaction stabilizes RAD51 monomers in a nucleation-competent conformation. Small molecules such as CAM833 disrupt this interface, impairing RAD51 assembly and reducing DNA damage–induced foci formation [35]. By targeting regulated assembly surfaces rather than the conserved ATPase core, such compounds may achieve improved selectivity.

### Mechanistic insights and translational leverage

Traditional descriptions of RAD51 emphasize strand exchange chemistry. However, in breast cancer, therapeutic response correlates less with catalytic activity and more with filament assembly state. The presence or absence of RAD51 nuclear foci predicts treatment sensitivity [12], whereas restoration of RAD51 loading is closely associated with acquired resistance [5, 12, 13]. Moreover, replication fork protection requires stabilization of RAD51 filaments and can occur independently of complete strand exchange proficiency [4, 6]. These findings indicate that the biologically relevant variable is filament assembly rather than catalytic turnover, shifting emphasis from enzymology to mediator-dependent assembly kinetics.

Structural understanding of RAD51 filament architecture clarifies why BRCA mutations sensitize tumors and why reversion mutations restore resistance [5, 12, 13]. Fork protection can persist despite partial HR defects [4, 6], and targeting mediator interfaces offers a rational strategy to disrupt adaptive repair buffering networks [36]. In this context, the RAD51 filament is not merely a molecular structure executing repair, but a functional unit of therapeutic adaptation under replication stress.

## Regulatory Architecture of RAD51: Mediator Networks, Cell-Cycle Control, and Checkpoint Integration

RAD51 does not function autonomously in mammalian cells. Its activity is embedded within a multilayered regulatory architecture integrating DNA damage sensing, chromatin remodeling, cell-cycle progression, and proteostatic control [37, 38]. This regulatory ecosystem determines whether RAD51 assembles productively at DSBs, stabilizes stalled replication forks, or remains inactive. In breast cancer, perturbations within this network frequently precede alterations in RAD51 catalytic function itself. Thus, tumor vulnerability or resistance often reflects shifts in regulatory balance rather than intrinsic recombinase defects [37, 39].

### The BRCA1–PALB2–BRCA2 axis: controlled filament nucleation

The BRCA pathway serves as the principal gatekeeper of RAD51 assembly [39, 40]. BRCA1 acts upstream of RAD51 loading by licensing DNA end resection through coordinated interactions with CtIP and the MRN complex, thereby generating the 3′ ssDNA substrate required for filament formation [41–43]. Simultaneously, BRCA1 antagonizes 53BP1-mediated end protection, biasing repair toward HR rather than non-homologous end joining [20, 44].

In BRCA1-deficient breast cancer, impaired resection limits RAD51 loading and produces HRD. However, loss of 53BP1 or Shieldin components restores resection and partially rescues RAD51 assembly [20, 21, 45]. These findings demonstrate that RAD51 deficiency often reflects regulatory imbalance rather than intrinsic recombinase incapacity.

Partner and localizer of BRCA2 (PALB2) functions as a structural bridge linking BRCA1 to BRCA2 [46]. Germline PALB2 mutations confer substantial breast cancer risk, underscoring that mediator-dependent control of RAD51 assembly is tumor suppressive [46, 47].

BRCA2 serves as the principal RAD51 chaperone. Compared with BRCA1, BRCA2 plays a more direct mechanistic role in RAD51 loading and stabilization at stalled and reversed replication forks. Consequently, BRCA2 deficiency produces particularly profound defects in RAD51 filament stabilization and replication fork protection. Through its BRC repeats and C-terminal RAD51-binding domain, BRCA2 delivers RAD51 monomers to RPA-coated ssDNA while preventing inappropriate filament formation on double-stranded DNA [22, 26, 48]. In BRCA2-deficient tumors, RAD51 loading is profoundly impaired, conferring PARP inhibitor sensitivity [9–11]. Conversely, restoration of BRCA2 function reinstates RAD51 foci and therapeutic resistance [5, 12, 13].

### RAD51 paralog complexes: stabilization and fine-tuning

Mammalian cells encode five RAD51 paralogs that assemble into two major complexes: RAD51B-RAD51C-RAD51D-XRCC2 (BCDX2) and RAD51C-XRCC3 (CX3) [49–51]. Although lacking strong strand exchange activity, these paralogs stabilize RAD51 filaments and facilitate efficient assembly. BCDX2 functions early during filament formation, promoting RPA displacement and stabilizing nucleation intermediates [49, 50]. Germline mutations in RAD51C and RAD51D are associated with hereditary breast and ovarian cancer [52, 53]. Emerging evidence further implicates paralogs in replication fork protection, extending their tumor suppressor role beyond classical DSB repair [51, 54]. The CX3 complex contributes to later recombination steps and intermediate processing [49, 50]. Dysfunction of paralog complexes often yields intermediate HR defects rather than complete deficiency, influencing therapy sensitivity in a context-dependent manner [39, 52].

### Checkpoint kinases and cell-cycle restriction

HR is largely restricted to S and G2 phases when sister chromatids are available as templates [37, 55]. Ataxia telangiectasia and Rad3-related (ATR)–checkpoint kinase 1 (CHK1) signaling at RPA-coated ssDNA coordinates replication stress responses and promotes RAD51 recruitment [56, 57]. In replication stress–high breast cancers, ATR signaling is frequently hyperactivated, generating co-dependency on checkpoint and RAD51-mediated repair pathways [39, 57]. CDK activity further regulates HR competence by controlling CtIP activation and BRCA1 phosphorylation [41, 58]. Disruption of this temporal coordination can increase aberrant recombination and genomic instability [37, 55].

### Post-translational regulation and chromatin context

RAD51 activity is additionally modulated by phosphorylation, ubiquitination, and chromatin accessibility [59, 60]. Cyclin dependent kinase (CDK)-dependent phosphorylation regulates HR timing [58], while checkpoint-mediated phosphorylation events modulate RAD51 recruitment and stability [59]. Helicase–ubiquitin ligases such as FBH1 act as anti-recombinases, limiting excessive filament stabilization [35, 60]. Loss of these regulatory brakes may enhance fork stabilization but increase genome instability. Chromatin context also shapes RAD51 activity. γH2AX spreading recruits chromatin remodelers [17, 61], and BRCA1 contributes to chromatin accessibility [62]. Epigenetic silencing of BRCA1 via promoter methylation can generate functional HRD without BRCA1 mutation [63].

### Regulatory failure in breast cancer

In breast cancer, RAD51 dysfunction most commonly arises from alterations in regulatory components rather than RAD51 mutation itself [5, 21, 39]. BRCA1/2 mutations, PALB2 defects, paralog alterations, Shieldin loss, and checkpoint rewiring dynamically shift RAD51 assembly capacity. Accordingly, RAD51 activity reflects a regulatory equilibrium rather than intrinsic enzymatic competence.

### Transition to a fork-centric perspective

Although the BRCA pathway classically governs RAD51 assembly at DSBs, increasing evidence indicates that fork-associated RAD51 activity is particularly consequential in therapy-resistant breast cancer [4, 6, 8, 57]. Restoration of mediator function re-establishes replication fork protection capacity under sustained replication stress, bridging DSB repair regulation to adaptive fork stabilization.

## Replication Fork Protection as the Central Adaptive Axis in Breast Cancer

The clinical framework for HR in breast cancer has traditionally emphasized DSB repair. BRCA1 and BRCA2 mutations impair RAD51 loading, producing HRD that sensitizes tumors to PARP inhibitors and platinum agents [9–11]. However, accumulating mechanistic and translational evidence indicates that RAD51-dependent replication fork protection, rather than classical strand exchange–mediated DSB repair alone, plays a decisive role in therapy resistance [4–6, 12, 13]. Recognizing this distinction reframes both biomarker development and therapeutic strategy.

### Replication stress as a defining feature of aggressive breast cancer

Aggressive breast cancers, particularly triple-negative breast cancer (TNBC), exhibit high levels of oncogene-driven replication stress [64–66]. MYC amplification, cyclin E overexpression, loss of gap 1 phase (G1) checkpoint control, and elevated proliferation rates generate persistent fork stalling and genome instability [64, 65]. In replication stress–high tumors, cellular viability depends less on repairing occasional breaks and more on stabilizing stalled and reversed replication intermediates [66].

### RAD51 at stalled forks: a function distinct from strand exchange

Upon replication stress, forks undergo reversal to form four-way junction–like structures [67–69]. RAD51 promotes fork reversal and, critically, protects nascent DNA from MRE11-mediated degradation [4, 6, 68]. This protective function requires filament formation but does not strictly require complete strand exchange, establishing a separation-of-function paradigm [4, 6, 7].

DNA translocases including SMARCAL1, ZRANB3, and HLTF cooperate with RAD51 in remodeling forks [67, 69]. When RAD51 stabilization fails, reversed forks become substrates for nucleolytic attack, leading to fork collapse and chromosomal instability [4, 6].

**Figure 2:**
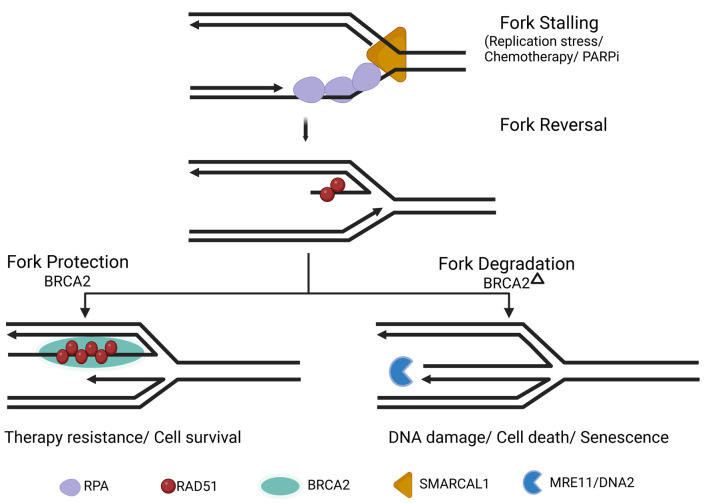
Replication fork remodeling and RAD51-mediated fork protection during replication stress. Replication stress induces fork stalling and reversal into a four-way junction structure. BRCA2 promotes RAD51 filament stabilization at reversed forks, protecting nascent DNA from MRE11/DNA2-mediated degradation and facilitating fork restart. In the absence of BRCA2/RAD51-mediated protection, reversed forks undergo nucleolytic degradation, contributing to genomic instability, DNA damage, and cell death.

This mechanistic uncoupling of fork protection from DSB repair provides a critical explanation for therapeutic resistance: fork stabilization can be restored even when full HR repair fidelity remains compromised [5, 12, 13]. Figure 2 illustrates the stepwise molecular events underlying replication fork remodeling, including fork stalling, fork reversal, RAD51 loading, fork protection versus nuclease-mediated degradation, and replication restart.

### BRCA2 and fork protection: a determinant of PARP inhibitor sensitivity

BRCA2-deficient cells display pronounced degradation of stalled replication forks due to impaired RAD51 filament stabilization [4]. This fork degradation contributes directly to PARP inhibitor sensitivity [6, 70]. Importantly, PARP inhibitors induce replication-associated DNA lesions and trap PARP1 on DNA, intensifying replication stress [71, 72]. Under these conditions, failure of fork protection exacerbates cytotoxicity. Restoration of fork stability, even without full correction of HR defects, can attenuate PARP inhibitor sensitivity [5, 12, 13]. This central role of BRCA2 in RAD51 filament stabilization may explain why BRCA2-deficient tumors often exhibit particularly pronounced replication fork instability under therapeutic stress.

### Resistance mechanisms converge on fork stabilization

In PARP inhibitor–resistant breast cancers, diverse genetic alterations converge on re-establishing replication fork protection [5, 12, 13, 73]. These mechanisms include BRCA reversion mutations that restore RAD51 loading [5], loss of 53BP1–Shieldin components that permit DNA end resection and renewed RAD51 assembly [20, 21], upregulation of RAD51 expression [74], and suppression of MRE11-dependent nucleolysis at stalled forks [68]. Importantly, even partial restoration of fork stability can be sufficient to confer resistance, despite incomplete recovery of DSB repair fidelity [5, 12, 13]. Together, these findings indicate that fork stabilization—rather than full HR restoration—represents the critical adaptive requirement under sustained therapeutic pressure.

### Fork protection versus strand exchange: distinct clinical consequences

Classical HRD leaves durable genomic scars detectable by mutational signatures and chromosomal alteration patterns [75]. In contrast, fork protection capacity can fluctuate dynamically. Tumors may retain genomic features of historical HRD while reacquiring RAD51 foci and fork stabilization during therapy [12]. This divergence explains why static genomic HRD scores may fail to predict response in relapsed disease [76]. Tumors that reacquire replication fork protection while retaining incomplete HR repair may therefore occupy an intermediate or functionally rewired HR state rather than strictly HR-deficient or HR-proficient categories. Functional assessment of RAD51 loading and replication fork integrity therefore becomes potentially important for capturing the biologically relevant repair state.

### RAD51 dependency under therapeutic pressure

As tumors adapt to chronic replication stress, survival may become increasingly reliant on RAD51-mediated replication fork stabilization [6, 57]. This progressive reliance may define a state of RAD51 dependency that arises during treatment rather than existing intrinsically in untreated disease. In this setting, RAD51 functions as a mediator of stress tolerance, sustaining replication continuity under persistent genotoxic pressure. Importantly, restoration of replication fork protection does not universally substitute for complete HR repair. Instead, fork stabilization appears particularly advantageous under conditions of sustained replication stress, partial retention of compensatory repair pathways, and continued therapeutic selection pressure [57, 66]. Therapeutic targeting of RAD51 in such contexts may therefore induce replication catastrophe, even when HR proficiency has been partially restored.

Breast cancer evolution under therapy can thus be conceptualized as a dynamic transition from RAD51 deficiency to RAD51 restoration and ultimately to RAD51 dependency. This fork-centric framework integrates replication stress signaling, structural recombinase biology, and mechanisms of clinical resistance into a unified model of adaptive tumor evolution.

### Clinical evidence supporting a fork-centric model

Clinical observations are consistent with a fork-centric framework. PARP inhibitor–resistant tumors frequently regain RAD51 foci [5, 12], resistance can occur without complete restoration of BRCA function [5], suppression of fork degradation correlates with reduced therapeutic sensitivity [4, 6, 77], and ATR inhibition shows enhanced efficacy in replication stress–high settings [53, 65]. Collectively, these findings indicate that modulation of replication stress tolerance, rather than DSB repair alone, shapes treatment outcome [58]. Recent translational studies further demonstrate that functional HR restoration—assessed by RAD51 nuclear foci—better predicts resistance than static BRCA mutation status [12, 66, 78].

### Implications for biomarker development

If replication fork protection represents a central determinant of resistance, biomarker strategies must move beyond static genomic assessments [60, 65].

Functional evaluation of RAD51 foci formation [12], measurement of replication stress signaling (γH2AX, pCHK1) [48, 52], and assessment of fork degradation capacity [4, 6] may more accurately represent tumor state.

Prospective integration of dynamic RAD51 assays into clinical trials is increasingly advocated [58, 66, 79]. Such approaches may outperform HRD scar-based classifiers in relapsed disease [60, 65].

### Therapeutic implications of the fork protection model

In tumors that have regained RAD51 loading or fork stabilization following PARP inhibitor exposure, direct disruption of RAD51 assembly may restore vulnerability [36, 65]. Combinatorial approaches are likely to be particularly effective. Concurrent inhibition of RAD51 and ATR may destabilize stalled forks while preventing checkpoint-mediated recovery [53, 65]. Preclinical models demonstrate that ATR inhibition disrupts rewired HR and fork protection in PARP inhibitor–resistant BRCA-deficient tumors [65]. Similarly, platinum chemotherapy induces replication-associated lesions whose tolerance depends on RAD51-mediated fork protection [6, 58]. Transient attenuation of RAD51 function during platinum exposure may therefore limit fork stabilization without requiring sustained systemic suppression. Targeting mediator-dependent interfaces rather than the conserved ATPase core remains a rational strategy to enhance selectivity [36].

### Conceptual model of breast cancer evolution under therapy

In early disease—particularly BRCA1/2-mutant tumors—impaired RAD51 loading may define HRD and confer marked sensitivity to PARP inhibition [9–11]. Under therapeutic pressure, tumors frequently acquire alterations that partially restore RAD51 assembly or replication fork stabilization [5, 12, 13]. In advanced or relapsed settings, continued replication stress may render tumor survival increasingly dependent on RAD51-mediated fork protection [6, 64]. Therapeutic strategy must therefore evolve from exploiting RAD51 deficiency to targeting RAD51 dependency. Functional RAD51 assessment becomes potentially important [12, 66].

## RAD51 Dependency Across Breast Cancer Subtypes

Elevated RAD51 expression correlates with aggressive tumor biology and poor survival in breast cancer cohorts [80, 81]. High RAD51 levels may reflect replication stress–adapted disease rather than simple repair proficiency.

### TNBC: a replication stress–high ecosystem

TNBC exhibits pronounced replication stress driven by MYC amplification, cyclin E overexpression, and chromosomal instability [56, 57]. ATR–CHK1 signaling is often elevated [48, 53]. BRCA1-mutant TNBC initially displays HRD and sensitivity to PARP inhibitors [9–11]. Resistance commonly emerges through RAD51 restoration or fork protection rewiring [5, 12, 13]. Even BRCA-wildtype TNBC frequently demonstrates high endogenous replication stress [56, 57], positioning relapsed TNBC as a rational context for RAD51-directed strategies [58, 65].

### BRCA1/2-mutant breast cancer: from vulnerability to adaptive dependence

Early BRCA-mutant tumors show absent RAD51 foci and genomic HRD scars [9–11, 60]. Under therapy, BRCA reversion mutations and resection pathway rewiring restore RAD51 foci and fork stability [5, 12, 13, 20]. Elevated BRCA2 gene expression has also been associated with aggressive and highly proliferative breast cancers [82]. This suggests that amplification of the BRCA–RAD51 axis may confer growth advantage under replication stress. In this resistant state, tumor survival may depend on RAD51-mediated fork stabilization [6, 64], supporting evaluation of RAD51-targeted approaches after PARP inhibitor progression.

### Hormone receptor–positive luminal breast cancer: conditional RAD51 dependency

Luminal breast cancers typically exhibit lower baseline genomic instability than TNBC. However, therapy-driven contexts—endocrine resistance, CDK4/6 inhibitor exposure, and MYC activation—can increase replication stress signaling [83–85]. In such settings, RAD51-mediated repair may become more critical for sustaining proliferation [57, 58]. Biomarker-defined subsets with elevated RAD51 foci or ATR activation may be susceptible to replication stress–targeted strategies [53, 65]. RAD51 dependency in luminal disease thus appears conditional and context-dependent rather than constitutive.

### HER2-amplified breast cancer: proliferative signaling and DNA damage response crosstalk

Human epidermal receptor 2 (HER2) amplification drives proliferative signaling via PI3K–AKT and MAPK pathways [63]. Increased replication demand may promote replication stress and engage DNA damage response (DDR) pathways [58]. During combined HER2-targeted therapy and chemotherapy, RAD51-mediated fork protection may contribute to tolerance. Functional stratification based on replication stress markers rather than subtype alone may identify subsets responsive to RAD51 disruption [58, 64].

### Biomarker-driven stratification: beyond molecular subtype

Molecular subtype classification alone is insufficient to define RAD51 dependency. A functional stratification framework incorporating assessment of RAD51 nuclear foci [12], replication stress markers such as γH2AX and phosphorylated CHK1 [48], evidence of BRCA reversion mutations [5], and prior PARP inhibitor exposure may more accurately capture the operative repair state of a tumor. Such an approach could distinguish between HR-deficient, PARP-sensitive disease; tumors that have restored RAD51 activity and are resistant yet potentially RAD51-dependent; and BRCA-wildtype tumors characterized by high replication stress that may be vulnerable to synthetic lethal combinations targeting replication stress tolerance pathways.

In advanced disease, this dynamic functional assessment is likely to outperform static genomic HRD scoring by reflecting the evolving repair capacity of tumors under therapeutic pressure [60, 66].

### Clinical trial implications

Clinical development of RAD51-directed strategies should be aligned with the functional state of the tumor rather than relying solely on historical genomic classification. In PARP inhibitor–resistant TNBC with restored RAD51 foci, evaluation of RAD51-targeted approaches may be warranted. In replication stress–high TNBC, combinatorial strategies such as concurrent RAD51 and ATR inhibition may be particularly rational [65]. In endocrine-resistant hormone receptor–positive (HR+) disease, therapeutic regimens could be designed to exploit therapy-induced replication stress and emerging RAD51 dependency. Across these contexts, the central question shifts from identifying HRD to determining whether a tumor has become reliant on RAD51-mediated replication stress tolerance [58, 65].

### Integrative model

RAD51 dependency evolves dynamically over the course of therapy. In early BRCA-mutant tumors, impaired RAD51 loading may define a state of HRD that confers marked therapeutic vulnerability [9–11]. Under treatment pressure, tumors frequently acquire alterations that restore RAD51 assembly [5, 12, 13]. In advanced disease, sustained replication stress may render survival increasingly dependent on RAD51-mediated fork stabilization [6, 64]. Accordingly, therapeutic strategies must transition from exploiting RAD51 deficiency in early disease to dismantling RAD51 dependency in resistant tumors.

## Therapeutic Targeting of RAD51: Strategies, Constraints, and the Future of Fork-Centric Precision Oncology

The clinical success of PARP inhibitors established HRD as a therapeutically actionable state in breast cancer [9–11]. However, resistance driven by restoration of RAD51 loading or replication fork protection [5, 6, 12, 13] has exposed the limitations of HRD as a static biomarker. Increasing evidence suggests that replication fork stabilization, rather than DSB repair alone, sustains tumor survival in therapy-exposed disease [4, 6, 64]. In this context, directly targeting RAD51 or its regulatory interfaces becomes a compelling translational objective [36, 65]. Yet RAD51 presents unique challenges: it is potentially important in proliferating normal tissues, functions as a cooperative nucleoprotein filament, and lacks deep catalytic pockets typical of classical enzyme targets [14, 22]. Therapeutic development must therefore focus on selective disruption of regulated filament assembly and stress-dependent fork protection rather than indiscriminate catalytic inhibition [36, 86].

### Direct RAD51 inhibition: assembly interfaces as drug targets

Structural analyses of RAD51 have revealed mechanistically distinct interaction surfaces that are amenable to selective pharmacologic intervention [14, 22]. Among these, the BRCA2–RAD51 interface is particularly attractive because it governs mediator-dependent nucleation and filament stabilization [22, 25–27]. Small molecules such as CAM833 disrupt this interaction by occupying the hydrophobic pocket engaged by BRCA2 BRC repeats, thereby impairing regulated filament assembly without directly targeting the conserved ATPase core [36, 86]. Preclinical studies demonstrate that such compounds reduce DNA damage–induced RAD51 foci formation, disrupt filament clustering, sensitize tumor cells to ionizing radiation, and enhance the activity of PARP inhibitors in BRCA-proficient settings [36, 86].

Additional recombinase inhibitors, including B02 derivatives, further validate the feasibility of pharmacologic interference with RAD51 function [24, 87]. Nonetheless, substantial optimization of potency, selectivity, and pharmacokinetic properties will be required before these approaches can be translated into clinical practice [86, 88].

### Targeting RAD51 in the context of resistance

The fork-centric model predicts that RAD51 inhibition may be most effective in tumors that have re-acquired RAD51 activity following BRCA reversion mutations [5, 89], restoration of DNA end resection capacity [20, 21, 65], or adaptive rewiring of replication stress pathways [65]. In these contexts, tumor survival becomes increasingly dependent on RAD51-mediated replication fork stabilization [6, 64], and disruption of this protection may precipitate replication catastrophe despite partial restoration of HR proficiency [4, 6].

This therapeutic strategy differs fundamentally from HRD-directed approaches, in which RAD51 deficiency is exploited. Instead, the objective is to target RAD51 restoration and emerging dependency in resistant disease [5, 12, 13].

### Combination strategies: collapsing adaptive networks

Given the central role of RAD51 in replication stress tolerance, monotherapy is unlikely to achieve durable responses. Instead, rational combination strategies aim to dismantle the adaptive repair networks that buffer replication-associated damage in resistant tumors. Reintroducing PARP inhibition alongside RAD51 suppression may destabilize replication fork protection in PARP inhibitor–resistant disease [6, 65]. Similarly, ATR inhibition has been shown to disrupt rewired HR and fork stabilization in PARP inhibitor–resistant BRCA-deficient tumors [65], and combined RAD51–ATR inhibition may further destabilize stalled forks, prevent checkpoint-mediated recovery, and drive replication stress beyond tolerable thresholds [52, 65].

Platinum chemotherapy induces replication-blocking DNA lesions whose tolerance depends on effective fork stabilization [6, 58]; therefore, transient attenuation of RAD51 during platinum exposure may enhance cytotoxicity by limiting the tumor’s capacity to protect replication intermediates. Collectively, these approaches are designed not to exploit static repair deficiencies, but to collapse adaptive replication stress buffering networks that sustain survival under therapeutic pressure [58].

### Therapeutic window and toxicity considerations

A central challenge in targeting RAD51 lies in preserving an acceptable therapeutic index, as RAD51 is indispensable for genome maintenance in normal proliferative tissues, including hematopoietic and intestinal compartments [1, 37]. Complete catalytic inhibition would therefore be poorly tolerated. Strategies to widen the therapeutic window include biomarker-driven patient selection to identify tumors with heightened RAD51 dependency [66], pulsed or schedule-dependent dosing coordinated with cytotoxic therapy, and selective disruption of mediator-dependent assembly interfaces rather than indiscriminate inhibition of the catalytic core [36]. Exploiting the elevated baseline replication stress characteristic of tumor cells may further provide a differential vulnerability relative to normal tissues [57]. Careful pharmacodynamic monitoring—such as assessment of RAD51 foci suppression and replication stress markers—will be potentially important in early-phase trials to balance efficacy with tolerability [66, 78].

### Functional biomarkers for clinical development

Successful clinical implementation of RAD51-directed strategies will depend on the development of robust functional biomarkers capable of capturing tumor dependency in real time. Quantification of RAD51 nuclear foci [12] provides a direct measure of recombinase assembly, while assessment of replication stress signaling markers such as γH2AX, RPA foci, and phosphorylated CHK1 [48, 52] offers complementary insight into the cellular stress landscape. Detection of BRCA reversion mutations through circulating tumor DNA analysis [5] and longitudinal sampling in resistant disease [66] may further refine patient selection and response monitoring. Functional RAD51 assays could therefore serve both as predictive biomarkers and as pharmacodynamic readouts of target engagement in early clinical trials [66, 78].

### Potential resistance to RAD51 inhibition

As with other targeted therapies, resistance to RAD51 inhibition is likely to emerge through adaptive rewiring of replication stress management pathways. Potential mechanisms include upregulation of alternative fork stabilization factors such as RADX [69], increased reliance on translesion synthesis polymerases [90], checkpoint pathway rewiring [65], and acquisition of mutations affecting drug-binding interfaces. Experience with PARP inhibitor resistance has demonstrated that replication fork stabilization pathways are highly plastic and capable of rapid adaptation [5, 6, 12, 13]. Accordingly, systematic preclinical mapping of potential escape routes will be potentially important for the rational design of durable combination strategies [58].

### Broader implications beyond breast cancer

Replication stress–dependent reliance on RAD51-mediated fork protection is unlikely to be restricted to breast cancer [58]. BRCA-associated ovarian cancers exhibit fork protection–mediated mechanisms of PARP inhibitor resistance [5], while pancreatic cancers frequently harbor HR alterations and display replication stress signatures [91]. In certain lung cancers, activation of the ATR–CHK1 axis reflects replication stress vulnerability that may intersect with RAD51-dependent repair pathways [92]. These observations suggest that fork-centric precision oncology may have broad applicability across malignancies in which replication stress tolerance, rather than DSB repair alone, determines therapeutic response [58, 93].

### Concluding perspective: RAD51 as a dynamic determinant of therapeutic evolution

RAD51 biology in breast cancer reflects a dynamic evolutionary trajectory rather than a static repair phenotype. During early tumorigenesis, impaired RAD51 loading—most commonly through disruption of the BRCA pathway—drives genomic instability while simultaneously creating therapeutic vulnerability [9–11]. Under sustained therapeutic pressure, tumors frequently restore RAD51 assembly, thereby converting an initial repair defect into a mechanism of resistance [5, 12, 13]. In advanced disease, continued replication stress may render survival increasingly dependent on RAD51-mediated fork stabilization [6, 64], transforming RAD51 from a lost tumor suppressive function into a mediator of adaptive survival.

Future precision oncology strategies must therefore integrate functional assessment of RAD51 activity, incorporation of replication fork protection metrics, rational stress-amplifying therapeutic combinations, and careful optimization of the therapeutic window. If replication fork stabilization represents an important adaptive mechanism in therapy-resistant disease, then disruption of RAD51-mediated protection may represent a promising future strategy for overcoming acquired resistance [58, 86].

## Data Availability

The authors declare that data supporting the findings of this study are available within the article.
